# Enhancing repeatability of follicle counting with deep learning reconstruction high-resolution MRI in PCOS patients

**DOI:** 10.1038/s41598-024-84812-3

**Published:** 2025-01-07

**Authors:** Renjie Yang, Yujie Zou, Liang Li, Weiyin Vivian Liu, Changsheng Liu, Zhi Wen, Yunfei Zha

**Affiliations:** 1 Department of Radiology, Renmin Hospital of Wuhan University, No. 238 Jiefang Road, Wuchang District, Wuhan, 430060 China; 2 Reproductive Medicine Center, Renmin Hospital of Wuhan University, Wuhan, 430060 China; 3 GE Healthcare, MR Research, Beijing, 100080 China

**Keywords:** Ovary, Polycystic ovary syndrome, High resolution, Deep learning, Magnetic resonance imaging, Reproductive disorders, Medical imaging

## Abstract

Follicle count, a pivotal metric in the adjunct diagnosis of polycystic ovary syndrome (PCOS), is often underestimated when assessed via transvaginal ultrasonography compared to MRI. Nevertheless, the repeatability of follicle counting using traditional MR images is still compromised by motion artifacts or inadequate spatial resolution. In this prospective study involving 22 PCOS patients, we employed periodically rotated overlapping parallel lines with enhanced reconstruction (PROPELLER) and single-shot fast spin-echo (SSFSE) T2-weighted sequences to suppress motion artifacts in high-resolution ovarian MRI. Additionally, deep learning (DL) reconstruction was utilized to compensate noise in SSFSE imaging. We compared the performance of DL reconstruction SSFSE (SSFSE-DL) images with conventional reconstruction SSFSE (SSFSE-C) and PROPELLER images in follicle detection, employing qualitative indices (blurring artifacts, subjective noise, and conspicuity of follicles) and the repeatability of follicle number per ovary (FNPO) assessment. Despite similar subjective noise between SSFSE-DL and PROPELLER as assessed by one observer, SSFSE-DL images outperformed SSFSE-C and PROPELLER images across all three qualitative indices, resulting in enhanced repeatability in FNPO assessment. These results highlighted the potential of DL reconstruction high-resolution SSFSE imaging as a more dependable method for identifying polycystic ovary, thus facilitating more accurate diagnosis of PCOS in future clinical practices.

## Introduction

**Fig. 1:**
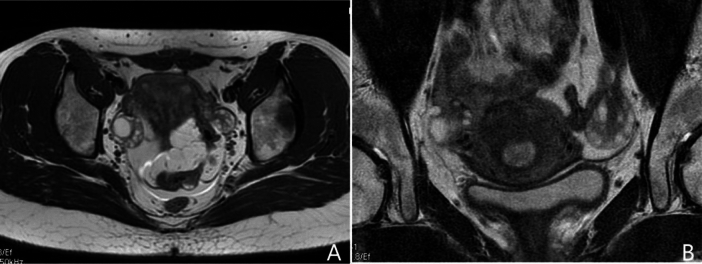
Current challenges in follicle counting using traditional fast spin-echo T2-weighted MR imaging. Panel (**A**) shows the full field of view image, which offers relatively high temporal resolution. However, its low spatial resolution makes follicle counting challenging. In contrast, panel (**B**) presents a small field of view image with higher spatial resolution, but it is prone to noise and motion artifacts due to its lower temporal resolution, which limits accurate follicle counting.

Polycystic ovary syndrome (PCOS), a prevalent reproductive disorder, impacts approximately 5–18% of women during their reproductive years^1,2^. The Rotterdam criteria^3^ outline the need to meet at least two specific criteria for diagnosing PCOS: 1) irregular menstruation (IM), 2) hyperandrogenism (HA), and 3) polycystic ovary (PCO). Currently, the assessment of PCO commonly relies on transvaginal ultrasonography (TVUS)^4,5^. However, the use of TVUS introduces significant variability of follicle count (FC) due to operator dependence and variations in transducer frequencies^3–6^. Additionally, TVUS is generally contraindicated in patients with no sexual history^7–9^. Consequently, ovarian MRI has emerged as a recommended alternative. Nonetheless, the depiction of follicles on traditional fast spin-echo (FSE) T2-weighted MR images continues to be compromised by motion artifacts or inadequate spatial resolution (Fig. 1), thereby reducing the repeatability of follicle counting^10,11^. As a result, there is an urgent requirement for motion-insensitive high-resolution MR images to enhance the repeatability of follicle counting.

To address motion artifacts in pelvic MRI, two principal approaches are commonly employed: (1) a radially oriented k-space filling pattern, such as periodically rotated overlapping parallel lines with enhanced reconstruction (PROPELLER) and BLADE^12–15^; and (2) an enhanced acquisition speed like single-shot FSE (SSFSE)^16,17^. In contrast to PROPELLER and BLADE, SSFSE demonstrates enhanced motion robustness but is less favored for high-resolution ovarian MRI due to its intrinsic blurring effect and relatively low signal-to-noise ratio (SNR)^18^.

In recent years, deep learning (DL) reconstruction has been increasingly employed across various anatomical regions to mitigate noise without prolonging scanning time^19–23^. The AIR Recon DL is a novel inline DL-based reconstruction algorithm that integrates a convolutional neural network (CNN) directly into the MRI reconstruction pipeline. The CNN was trained using supervised learning with paired near-perfect (high-resolution, low noise) and conventional (lower resolution, higher noise) MRI images. The training set included diverse anatomical images and was augmented with transformations such as rotations, flips, intensity gradients, phase shifts, and Gaussian noise to enhance robustness. The training database comprised 4 million image/augmentation pairs, with training conducted over a single epoch of 4 million iterations. The adaptive moment estimation optimizer was used to minimize the loss between predicted and reference images, avoiding the use of generative adversarial networks to prevent the generation of spurious features^24^. Consequently, it facilitates the reconstruction of images characterized by both high resolution and high SNR^24,25^. Despite these advancements, there are few studies^18,26^ investigating the impact of integrating high-resolution SSFSE imaging with DL reconstruction on the assessment of ovarian morphology. These studies have confirmed the benefits of DL reconstruction SSFSE T2WI in enhancing follicle visualization compared to conventional FSE T2WI. However, whether DL reconstruction SSFSE T2WI can also surpass PROPELLER T2WI remains uncertain. Therefore, the primary objective of the study is to assess the effectiveness of DL reconstruction SSFSE images in consistent follicle counting through a comprehensive comparison with conventional SSFSE and PROPELLER images.

## Materials and methods

### Study population

This prospective study obtained approval from the Clinical Ethics Committee of Renmin Hospital of Wuhan University (WDRY2021-K028) and strictly adhered to ethical principles in accordance with the Declaration of Helsinki. From January 2023 to September 2023, we prospectively recruited a total of 24 volunteers diagnosed with PCOS, comprising 4 adolescent girls and 20 adult women, who underwent ovarian MRI at our institution. For adult women, a PCOS diagnosis required a minimum of two of the following signs: IM, HA, and PCO. For adolescent girls, both IM and HA were required^3^. Informed consent was obtained from adult patients (18 years or older), while patients under 18 years of age provided parental consent alongside patient assent. Inclusion criteria encompassed the following: (1) age between 14 and 35 years; (2) presence of any PCOS phenotype as clinically confirmed; (3) absence of previous ovarian surgery; (4) no contraindications for undergoing MRI examination; and (5) non-pregnant status. Exclusion criteria were defined as follows: (1) unable to complete the MRI procedure; (2) significant magnetic susceptibility artifacts hindering follicle visualization; and (3) presence of ovarian lesions hindering follicle visualization.

### MRI acquisitions

Ovarian MRI was performed using a 3-T MR imaging unit (SIGNA Architect; GE Healthcare) equipped with a 30-channel phased-array coil. The imaging protocol involved acquiring axial, coronal, and sagittal planes for both PROPELLER T2-weighted sequences (TR/effective TE = 5358–7240 ms/85 ms; receiver bandwidth =  ± 62.5 kHz; echo train length = 32; number of excitations = 3; resolution = 0.5 × 0.5 × 3.0 mm^3^; and total acquisition time = 7 min 12 s–7 min 20 s) and SSFSE T2-weighted sequences (TR/effective TE = 1700 ms/84 ms; receiver bandwidth =  ± 62.5 kHz; number of excitations = 1; resolution = 0.5 × 0.5 × 3.0 mm^3^; and total acquisition time = 1 min 34 s–2 min 5 s). Notably, breath holding was not required during the acquisition process. The MRI examinations were all performed by a radiology technologist (R.Y.) with 20 years of experience in pelvic MR imaging.

### Image reconstruction

For the PROPELLER sequences, conventional reconstruction was used. For the SSFSE sequences, both conventional and DL reconstructions were employed, resulting in SSFSE-C and SSFSE-DL images, respectively. The AIR Recon DL (DV29.1_R04, GE Healthcare, USA) algorithm is integrated into the image reconstruction pipeline and enables the generation of two distinct image sets from a single raw k-space dataset^24^. It offers three denoising levels (low, medium, and high), with the high denoising level selected for this study. Notably, the AIR Recon DL algorithm was entirely trained by the vendor (GE Healthcare), and no data from our institution were used in its development. This study was conducted without additional training of the DL reconstruction algorithm. Finally, each participant contributed three distinct image datasets: SSFSE-C, SSFSE-DL, and PROPELLER.

### Qualitative image analysis

All MR image datasets were transferred to the picture archiving and communication system (PACS). Subsequently, the axial images of SSFSE-C, SSFSE-DL, and PROPELLER for each patient were selected for qualitative assessment. All the image sets were presented in a random order and evaluated independently by observer 1 (L.L., 13 year’s experience in abdominal MRI diagnostics) and observer 2 (Z.W., 4 year’s experience in abdominal MRI diagnostics) without knowledge of the clinical and sequence information.

**Fig. 2:**
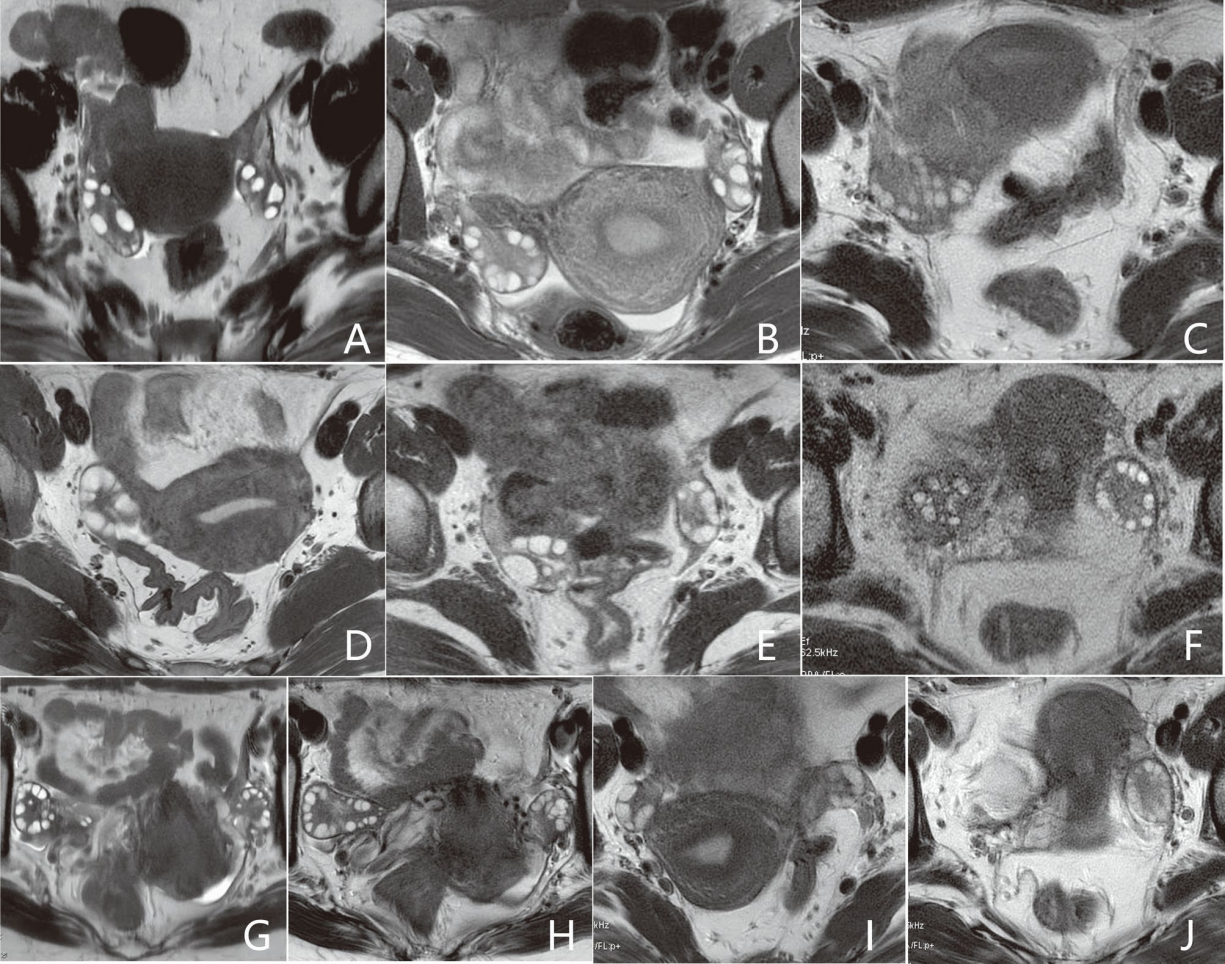
Scoring criteria for qualitative analysis. Scores of 3, 2, and 1 for blurring artifacts are assigned to (**A**–**C**), respectively. Scores of 3, 2, and 1 for subjective noise are assigned to (**D**–**F**), respectively. Scores of 4, 3, 2, and 1 for conspicuity of follicles are assigned to (**G**–**J**), respectively. Higher scores indicate better image quality.

The assessment criteria included the evaluation of blurring artifacts on a 3-point scale (3 = almost no visible blurring artifacts, 2 = blurring present but not interfering with the visualization of follicles, 1 = blurring present and interfering with the visualization of follicles), subjective noise on a 3-point scale (3 = almost no visible noise, 2 = noise present but not interfering with the visualization of follicles, 1 = noise present and interfering with the visualization of follicles), and the conspicuity of follicles scored on a 4-point scale (4 = excellent, almost no visible artifacts with clear delineation of follicles; 3 = good, slight artifacts but without impairing follicle counting; 2 = moderate, moderate artifacts with partial impairment of follicle counting; 1 = poor, severe artifacts with unrecognizable delineation of follicles). Refer to Fig. 2 for illustration.

### Follicle counting

To determine the follicle number per ovary (FNPO), follicles falling within the 2–9 mm range in the long axis were meticulously identified and counted on axial MR images. To minimize potential errors arising from the complex three-dimensional structure of the ovaries, cross-referencing with corresponding sagittal and coronal images was performed.

Each patient’s FNPO for bilateral ovaries underwent a thorough evaluation process. Observer 1 conducted two separate assessments, ensuring consistency and reliability within their observations. To address any potential recall bias, the second assessment was conducted after a 4-week interval. This interval was strategically chosen to strengthen the reliability and validity of subsequent evaluations. Furthermore, to assess agreement between observers, observer 2 independently evaluated the FNPO for each patient. Prior to conducting the formal image analysis and follicle counting, the two observers underwent a short training session.

### Statistical analysis

Initial assessments of normality and homogeneity of variance were conducted using the Kolmogorov–Smirnov and Levene tests, respectively. Wilcoxon signed-rank tests were employed to compare qualitative results between SSFSE-DL and SSFSE-C, as well as between SSFSE-DL and PROPELLER. Inter-observer agreement was assessed using Cohen’s weighted kappa, with 95% confidence intervals (CIs) calculated.

Intra-observer and inter-observer repeatability for FNPO assessment with each image type were evaluated using intraclass correlation coefficients (ICCs). The ICCs were categorized as indicating almost perfect agreement (0.81–1.00), substantial agreement (0.61–0.80), moderate agreement (0.41–0.60), fair agreement (0.21–0.40), and poor agreement (0.00–0.20). Bland‒Altman plots were generated to visually represent intra-observer or inter-observer variability, illustrating the mean differences and 95% limits of agreement (LOA). Paired *t* tests were conducted to assess the significance of intra-observer and inter-observer variations for SSFSE-DL compared to those for SSFSE-C and PROPELLER. Additionally, paired *t*-tests were employed to compare intra-observer and inter-observer variations within each image type. Statistical analyses were performed using MedCalc 11.6 software, with a predefined significance level set at *P* < 0.05.

## Results

### Patient characteristics

Out of the initially recruited cohort of 24 patients, two adult women were excluded from the study. One patient was unable to complete the examination due to claustrophobia, while the other was excluded due to the presence of an ovarian serous cystadenoma on the left ovary. As a result, a total of 22 patients were included in the study, comprising 4 adolescent girls and 18 adult women. Throughout the study, a comprehensive evaluation was conducted on a total of 44 ovaries. Detailed demographic characteristics of the patients can be found in Table 1.

**Table 1:** Patient demographics.

Characteristics	Value
Study patients, n	22
Adolescent girls (< 18 years), n (%)	4 (18%)
Adult women (≥ 18 years), n (%)	18 (82%)
Age, mean ± SD (range), year	22.3 ± 4.9 (15–32)
Gynecological age, mean ± SD (range), year	10.4 ± 4.7 ( 3–21)
BMI, mean ± SD (range), kg/m2	24.0 ± 3.4 (16.1–32.4)

*SD* standard deviation, *BMI* body mass index.

### Qualitative image analysis

**Fig. 3:**
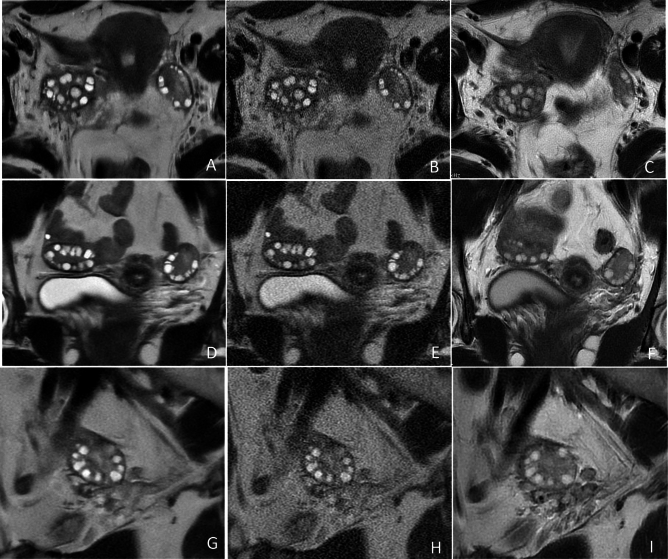
Representative T2-weighted MR images of the ovaries in a 17-year-old adolescent girl with PCOS. The SSFSE-DL images (**A**–**G**) clearly depict bilaterally enlarged ovaries and an increased number of peripheral follicles with minimal noise and blurring artifacts, providing the best conspicuity of follicles. The use of AIR Recon DL enhances the contrast between follicles and the surrounding ovarian stroma. The conspicuity of follicles in the SSFSE-C images (**B**, **E**, **H**) is mainly affected by noise, while that in the PROPELLER images (**C**, **F**, **I**) is primarily impaired by motion-related blurring artifacts.

**Fig. 4:**
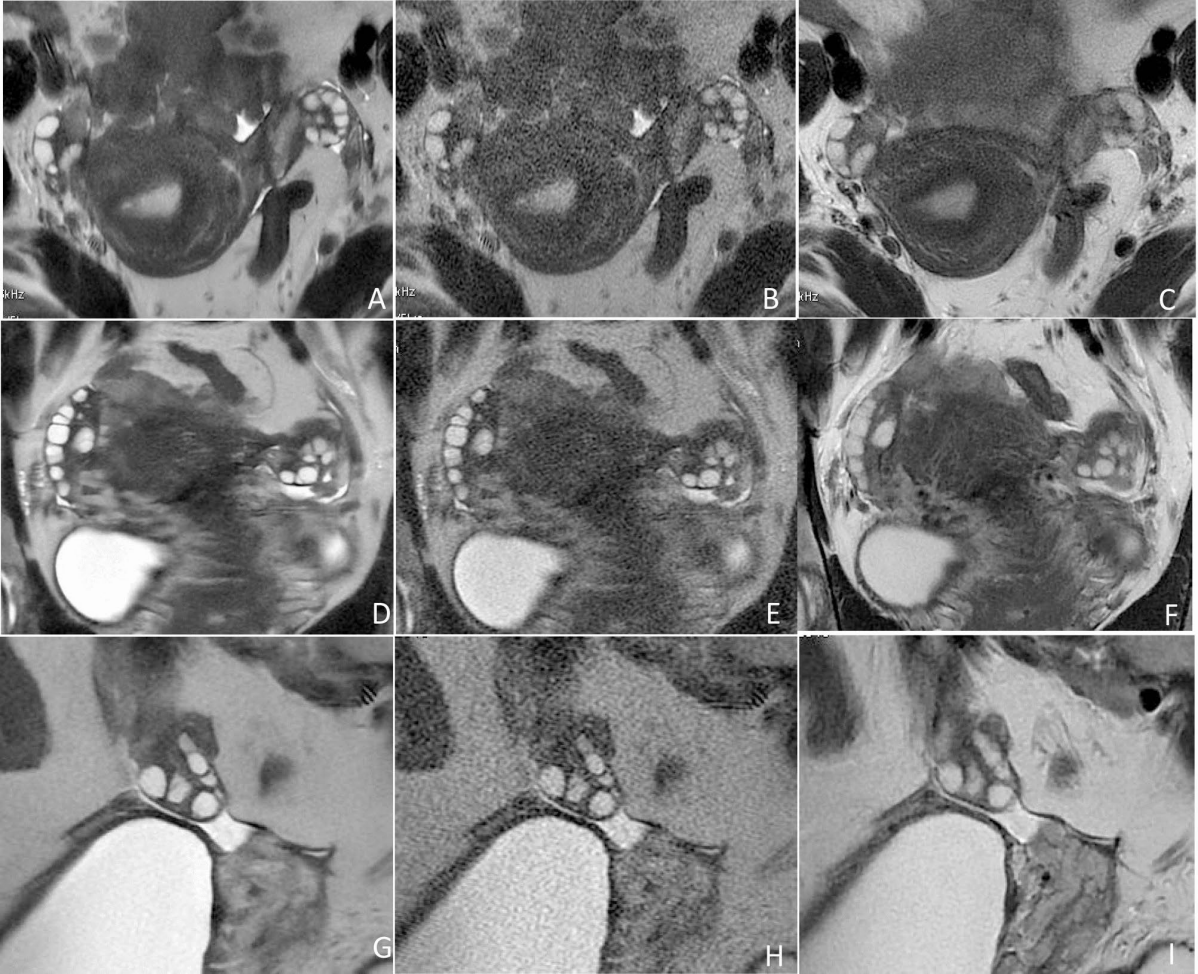
Representative T2-weighted MR images of the ovaries in a 24-year-old woman with PCOS. The SSFSE-DL images (**A**, **D**, **G**) clearly show bilateral ovaries and follicles with minimal noise and blurring artifacts, providing optimal conspicuity of follicles. In SSFSE-C images (**B**, **E**, **H**), follicle conspicuity is primarily influenced by noise, whereas in PROPELLER images (**C**, **F**, **I**), it is predominantly affected by motion-related blurring artifacts.

The results of the qualitative image analyses are presented in Table 2. Both observers reported significant improvements in blurring artifacts, subjective noise, and conspicuity of follicles on SSFSE-DL compared to those on SSFSE-C (*P* < 0.05). Notably, observer 2 reported higher scores with significant differences for the three qualitative indicators with SSFSE-DL than with PROPELLER (*P* < 0.05). However, the scores for subjective noise provided by observer 1 did not significantly differ between SSFSE-DL and PROPELLER. Two representative cases are illustrated in Figs. 3 and 4. The inter-observer agreements for the three qualitative items were moderate to good for SSFSE-DL (kappa, 0.575–0.723), SSFSE-C (kappa, 0.480–0.713), and PROPELLER (kappa, 0.588–0.725), respectively.

**Table 2:** Results of the qualitative image analyses.

Items	Observer	SSFSE-DL	SSFSE-C	PROPELLER	SSFSE-DL versus SSFSE-C	SSFSE-DL versus PROPELLER
Blurring artifacts	1	2.546 ± 0.510	2.136 ± 0.640	2.091 ± 0.684	***P* = 0.004**	***P* < 0.001**
	2	2.591 ± 0.503	2.092 ± 0.610	2.046 ± 0.785	***P* < 0.001**	***P* < 0.001**
Kappa (95% CI)		0.723 (0.433–1.000)	0.480 (0.165–0.794)	0.588 (0.324–0.852)		
Subjective noise	1	2.773 ± 0.429	1.364 ± 0.492	2.636 ± 0.492	***P* < 0.001**	*P* = 0.186
	2	2.727 ± 0.456	1.409 ± 0.503	2.409 ± 0.590	***P* < 0.001**	***P* = 0.048**
Kappa (95% CI)		0.637 (0.265–1.000)	0.713 (0.413–1.000)	0.593 (0.321–0.864)		
Conspicuity of follicles	1	3.455 ± 0.671	2.500 ± 0.673	2.136 ± 0.710	***P* < 0.001**	***P* < 0.001**
	2	3.227 ± 0.752	2.636 ± 0.727	2.364 ± 0.848	***P* = 0.012**	***P* = 0.001**
Kappa (95% CI)		0.575 (0.303–0.846)	0.547 (0.274–0.820)	0.725 (0.529–0.921)		

The scores for the qualitative items are presented as mean ± standard deviation, with higher scores indicating better outcomes. Blurring artifacts and subjective noise are scored on a 3-point scale, while follicle conspicuity is scored on a 4-point scale. The scores were compared using the Wilcoxon signed-rank test, and results with *P*-values < 0.05 are highlighted in bold. Cohen’s weighted kappa (95% CI) values for inter-observer agreement are shown: 0.81–1.00 (excellent), 0.61–0.80 (good), 0.41–0.60 (moderate), 0.21–0.40 (fair), and < 0.20 (poor). *SSFSE-C* single-shot fast spin-echo acquisition using conventional reconstruction; *SSFSE-DL* single-shot fast spin-echo acquisition using deep learning reconstruction; *PROPELLER* periodically rotated overlapping parallel lines with enhanced reconstruction; *CI* confidence intervals.

### Repeatability of follicle counting

**Fig. 5:**
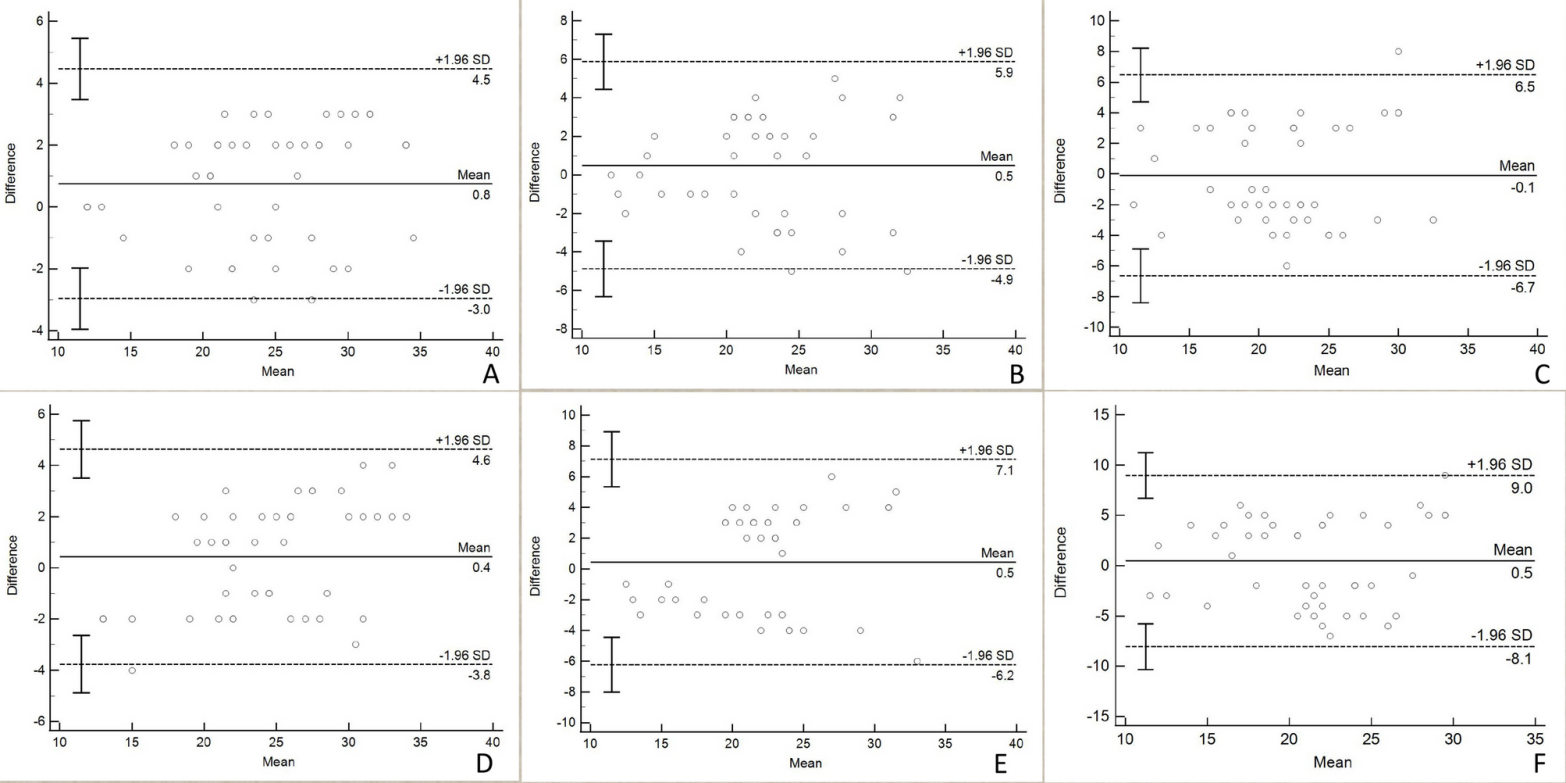
Bland‒Altman plots for intra-observer and inter-observer differences of FNPO assessment. The 95% limits of agreement (LOA) for intra-observer (**A**) and inter-observer (**B**) variability on SSFSE-DL images are narrower than the those for intra-observer (**C**) and inter-observer (**D**) variability on SSFSE-C images, as well as the intra-observer (**E**) and inter-observer (**F**) variability on PROPELLER images.

Descriptive statistics for the FNPO assessment of 44 ovaries are presented in Table 3. The FNPO assessed by the two observers ranged from 10 to 36. Intra-observer and inter-observer ICCs for FNPO assessment across the three image types are shown in Table 4. SSFSE-DL exhibited both higher intra-observer and inter-observer ICCs (0.938 and 0.922, respectively) compared to SSFSE-C (0.873 and 0.794, respectively) and PROPELLER (0.803 and 0.662, respectively). Bland–Altman plots depict the mean differences and 95% LOAs for intra-observer and inter-observer variability in FNPO assessment based on each image type (Fig. 5). The intra-observer 95% LOAs for the SSFSE-DL, SSFSE-C, and PROPELLER images ranged from − 3.0 to 4.5, − 4.9 to 5.9, and − 6.7 to 6.5, respectively, while the inter-observer 95% LOAs ranged from − 3.8 to 4.6, − 6.2 to 7.1, and − 8.1 to 9.0, respectively. SSFSE-DL exhibited narrower 95% LOAs compared to SSFSE-C and PROPELLER. Table 5 presents comparisons of the intra-observer and inter-observer absolute differences in the FNPO assessment. The results indicate that SSFSE-DL demonstrated significantly lower intra-observer and inter-observer absolute differences compared to both SSFSE-C and PROPELLER (*P* < 0.05). Additionally, no statistically significant differences were observed (*P* > 0.05) between the intra-observer and inter-observer absolute differences for SSFSE-DL. In contrast, for SSFSE-C and PROPELLER, the intra-observer absolute differences were significantly lower than the inter-observer absolute differences (*P* < 0.05).

**Table 3:** Descriptive statistics for FNPO.

Image type	Observer	Minimum	Maximum	Mean	SD
SSFSE-DL	1	12	35	24.7	5.9
	2	14	33	24.3	5.0
	1*	12	35	24.0	5.5
SSFSE-C	1	12	34	22.7	5.5
	2	13	36	22.2	5.1
	1*	12	35	22.2	5.4
PROPELLER	1	10	34	21.5	5.4
	2	11	29	21.1	5.1
	1*	10	34	21.6	5.2

*FNPO* follicle number per ovary, *SD* standard deviation, *SSFSE-C* single-shot fast spin-echo acquisition using conventional reconstruction, *SSFSE-DL* single-shot fast spin-echo acquisition using deep learning reconstruction, *PROPELLER* periodically rotated overlapping parallel lines with enhanced reconstruction; *The second assessment by observer 1.

**Table 4:** Repeatability of FNPO assessment for three image types.

Image type	Intra-observer ICC (95% CI)	Inter-observer ICC (95% CI)
SSFSE-DL	0.938 (0.879–0.967)	0.922 (0.861–0.956)
SSFSE-C	0.873 (0.780–0.928)	0.794 (0.653–0.882)
PROPELLER	0.803 (0.665–0.887)	0.662 (0.457–0.800)

ICC values are presented as mean (95% confidence intervals). *FNPO* follicle number per ovary; *ICC* intraclass correlation coefficient; *CI* confidence intervals, *SSFSE-C* single-shot fast spin-echo acquisition using conventional reconstruction; *SSFSE-DL* single-shot fast spin-echo acquisition using deep learning reconstruction; *PROPELLER* periodically rotated overlapping parallel lines with enhanced reconstruction.

**Table 5:** Comparisons of intra-observer and inter-observer absolute differences in FNPO assessment.

Parameter	SSFSE-DL	SSFSE-C	PROPELLER	SSFSE-DL versus SSFSE-C	SSFSE-DL versus PROPELLER
Intra-observer difference	1.796 ± 0.930 (1.513–2.078)	2.455 ± 1.266 (2.070–2.839)	3.046 ± 1.311 (2.647–3.444)	***P* = 0.002**	***P* < 0.001**
Inter-observer difference	2.023 ± 0.821 (1.773–2.272)	3.182 ± 1.206 (2.815–3.549)*	4.000 ± 1.643 (3.501–4.499)*	***P* < 0.001**	***P* < 0.001**

Absolute differences are presented as mean ± standard deviation, with 95% confidence intervals in parentheses. Comparisons were conducted using paired *t* tests, and results with *P*-values below 0.05 were emphasized in bold. *FNPO* follicle number per ovary, *SSFSE-C* single-shot fast spin-echo acquisition using conventional reconstruction, *SSFSE-DL* single-shot fast spin-echo acquisition using deep learning reconstruction, *PROPELLER* periodically rotated overlapping parallel lines with enhanced reconstruction; *Statistically significant between the intra-observer and inter-observer absolute differences. Significant values are in bold.

## Discussion

This study represents a pioneering effort in evaluating the repeatability of follicle counting using SSFSE-DL and PROPELLER T2-weighted images. Our findings emphasize the substantial improvement in follicle visualization and the facilitation of highly repeatable follicle counting achieved with SSFSE-DL images.

Spatial resolution in MR images is crucial for accurate follicle detection. To enhance follicle visualization, we employed SSFSE and PROPELLER T2WI sequences with a high spatial resolution of 0.5 × 0.5 × 3.0 mm^3^. However, the advantageous high spatial resolution is susceptible to introducing image noise, especially when utilizing the conventional inverse Fourier transform (iFT) reconstruction algorithm^24^. Consequently, severe noise affected follicle conspicuity on SSFSE-C images, consistent with the findings of Brown et al.^9^, who reported notable noise in 3 mm SSFSE images even when the slice thickness was increased to 6 mm. For the PROPELLER images, increasing the number of excitations to three effectively suppressed the noise but came at the cost of sacrificing scanning time. In contrast, the introduction of AIR Recon DL resulted in a significant reduction of noise in SSFSE-DL images without compromising temporal resolution. AIR Recon DL leverages a deep convolutional neural network model with more than four million unique pattern recognitions for noise, thus facilitating image reconstruction with a higher SNR without the need for additional scanning parameter optimization^24^. This improved SNR highlighted the contrast between follicles and the surrounding ovarian stroma in SSFSE-DL images, enabling a clearer delineation of follicles.

In addition to spatial resolution, motion artifacts represent another factor influencing follicle display. BLADE, a specialized technique for motion artifact suppression, was confirmed to improve the depiction of uterine and ovarian morphology compared with the conventional FSE sequences^12,13^. However, our study revealed that PROPELLER (similar to BLADE) exhibited more pronounced blurring artifacts in the ovaries than did SSFSE-DL, consistent with Tsuboyama et al.’s findings^17^, emphasizing the superior image quality of the uterus in SSFSE-DL. In our study, the total examination time with PROPELLER sequences was approximately 70% longer than that with SSFSE sequences. The prolonged scanning time of PROPELLER sequences exacerbated challenges related to respiratory motion and intestinal peristalsis^27^.

Therefore, temporal resolution emerges as a crucial factor in ovarian MRI. The SSFSE sequence, capable of acquiring a slice in less than a second, provides high temporal resolution, effectively freezing motion related to respiratory and intestinal peristalsis. However, compared to SSFSE-DL images, SSFSE-C images displayed more pronounced blurring artifacts in the ovaries due to the iFT algorithm’s partial removal of Gibbs ringing artifacts, leading to resolution degradation and blurring^24^. In contrast, AIR Recon DL improves image sharpness by interpolating high-frequency data^24^, thereby enhancing follicle conspicuity.

The precise quantification of follicles is important in identifying PCO in individuals suspected of having PCOS, especially considering the well-established superiority of follicle count as a more sensitive indicator compared to ovarian volume^28,29^. Currently, TVUS is the most widely employed imaging modality for follicle detection^5,6^. However, Nylander et al.^30^ conducted a study revealing that follicle counts ≤ 9 mm, as detected by TVUS, were approximately 20% smaller than those identified by 2D MRI in overweight women with PCOS. Similarly, another study^31^ reported that TVUS-detected follicle counts at sizes ≤ 9 mm were approximately 6 fewer than those identified by 2D MRI in women with infertility. Despite the improved ability to detect follicles, conventional 2D FSE MRI sequences only showed poor to moderate inter-observer agreement in follicle counting when using an acquisition voxel size exceeding 0.7 × 0.7 × 3.0 mm^3^^29,32^. This compromised repeatability in follicle counting emanates from the reduced visibility of follicles in conventional FSE T2-weighted images^10,11^. Leveraging the enhanced conspicuity of follicles, SSFSE-DL exhibited superior repeatability in FNPO assessment compared to SSFSE-C and PROPELLER. This enhancement was substantiated by higher ICCs, narrower LOAs, and lower intra-observer and inter-observer absolute differences. Moreover, the comparable absolute differences between inter-observer and intra-observer assessments with SSFSE-DL suggested the potential for consistent evaluations by different observers. Conversely, employing PROPELLER with two different observers during FNPO assessment might introduce elevated inter-observer variability, which could impact the identification of PCO. Therefore, SSFSE-DL, boasting high temporal and spatial resolution, effectively addressed the poor repeatability associated with conventional MRI sequences for follicle counting, thereby showcasing remarkable follicle detection capabilities.

Nevertheless, several limitations persist in our study. First, we included both adolescent girls and adults with PCOS, omitting control subjects. This design focused our investigation on assessing the repeatability of follicle counting rather than establishing definitive PCO identification thresholds. It is essential to note that ensuring the repeatability of a novel imaging method is a fundamental step before defining reliable PCO thresholds. Second, we did not group follicles by size due to challenges in accurately segmenting follicle sizes across the different image types, each offering varying levels of clarity. This could have introduced errors in the grouping process. Therefore, we focused on the 2–9 mm follicle size range, which is most important for PCO identification, to ensure reliable comparative results. Third, the absence of a comparison involving DL-reconstructed PROPELLER was due to the unavailability and uncertainty surrounding PROPELLER images using AIR Recon DL. Future investigations are imperative to compare DL-reconstructed PROPELLER and SSFSE images. Fourth, although SSFSE-DL enhances the quality of follicle images in this study, follicle detection remains time-consuming and observer-dependent, as it is still performed manually. However, when combined with a similar composite network for automatic detection, as reported by Li et al.^33^, this approach has the potential to further improve both the efficiency and repeatability of follicle counting. This will be a key focus of our future research.

In conclusion, the application of DL reconstruction high-resolution SSFSE markedly enhanced follicle visualization compared to PROPELLER, resulting in improved repeatability in follicle counting for PCOS patients. Therefore, it could serve as a more dependable imaging method for identifying PCO, thus facilitating more accurate diagnosis of PCOS in future clinical practices.

## Data Availability

The datasets generated during and/or analyzed in the current study can be obtained from the corresponding author on reasonable request.
